# Multiple Congenital Colonic Stenosis: A Rare Gastrointestinal Malformation

**DOI:** 10.1155/2016/6329793

**Published:** 2016-03-15

**Authors:** Zambaiti Elisa, Chiaramonte Cinzia, Salerno Sergio, Li Voti Giuseppe, Siracusa Fortunato

**Affiliations:** ^1^Pediatric Surgical Unit, Department of Mother and Child Care, AOU Policlinico Paolo Giaccone, Via Giordano 3, 90127 Palermo, Italy; ^2^Section of Radiological Sciences, Department of Radiology, AOU Policlinico Paolo Giaccone, Via del Vespro 127, 90127 Palermo, Italy

## Abstract

Congenital colonic stenosis is a rare pediatric condition. Since 1968, only 16 cases have been reported in the literature. To the authors' knowledge, multiple congenital colonic stenosis has not been previously reported in the literature. We report the case of a 2-month-old male, presented at our Neonatal Intensive Care Unit with a suspicion of intestinal malrotation. Clinical examination revealed persistent abdominal distension. During the enema examination, the contrast medium appeared to fill the lumen of the colon up to three stenotic segments and could not proceed further. Intraoperatively we confirmed the presence of four types of colonic atresia, located in the ascending, transverse, and descending colon, respectively, plus appendix atresia. First surgical steps consisted in resection of proximal stenotic segment, appendix removal, proximal cecostomy, and distal colostomy on ascending colon in order to preserve colonic length. Histopathological examination confirmed the diagnosis of colonic stenosis. Final surgical step consisted in multiple colocolostomy and enteroplasty. A planned two-stage procedure, consisting of resection with colostomy for decompression as the first step and a later anastomosis, is recommended in order to allow bowel length preservation.

## 1. Introduction

Congenital malformations affecting the colon are rare pediatric conditions often presenting as obstruction. Colonic atresia accounts for 5–10% of atresia in newborns [1]; stenosis is even more rare. The real incidence of congenital colonic stenosis (CCS) is not readily available because most cases of stenosis are acquired. Since 1968, only 16 cases of CCS have been reported in the literature [2–14]. Because of the rarity of the disease, little is known about this uncommon condition and management is still controversial. We present a case of multiple congenital colonic stenosis and review the literature with a special focus on the management of CCS. To the authors' knowledge, multiple congenital colonic stenosis has not been reported previously in the literature.

## 2. Case Report

An otherwise healthy male baby was referred to our Neonatal Intensive Care Unit at 2 months of life with a suspicion of intestinal malrotation. Being born at 35 + 4 weeks' gestation, with weight 2560 g, and with negative prenatal diagnosis, APGAR score was 9 at 1 and 5 minutes. The mother was a heavy smoker. Maternal and neonatal serology was negative.

**Figure 1:**
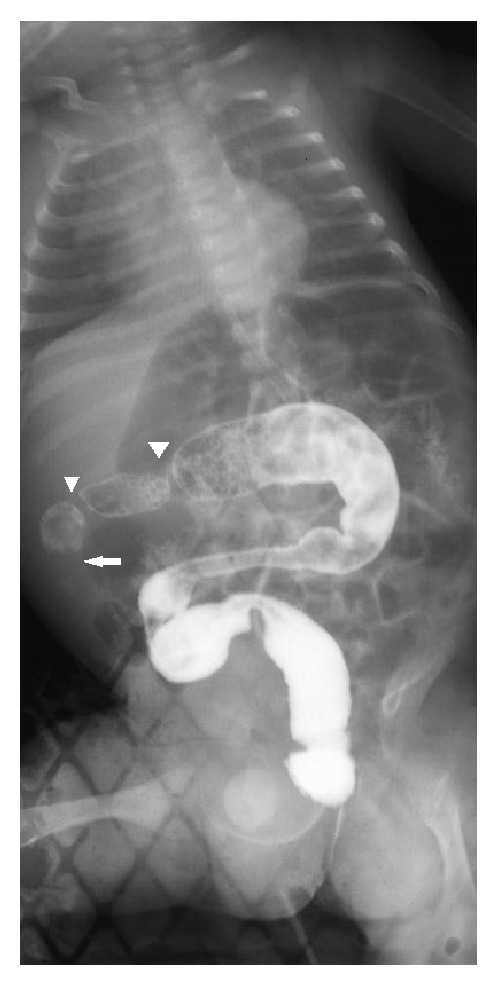


On arrival at our institute, clinical examination revealed a distended, quite painful abdomen with difficult feeding and intermittent vomit. At enema, the contrast medium appeared to fill the lumen of the colon up to three stenotic segments and could not proceed further, even with high pressure (Figure 1).

**Figure 2:**
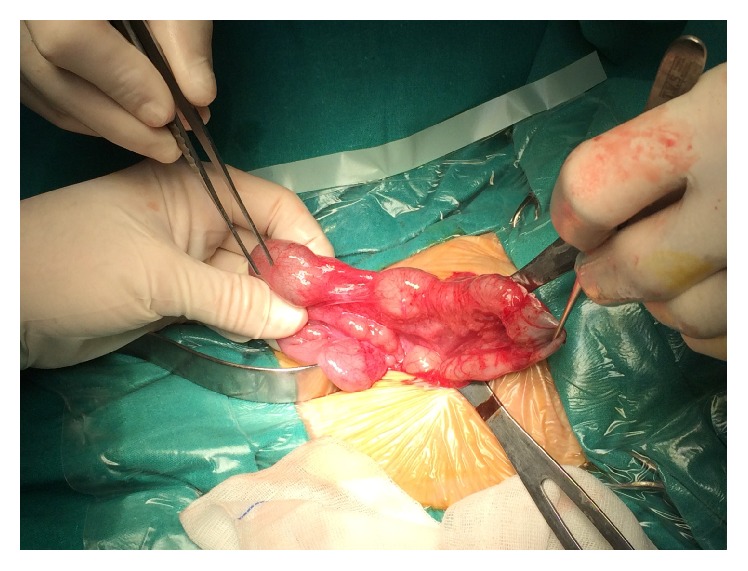


**Figure 3:**
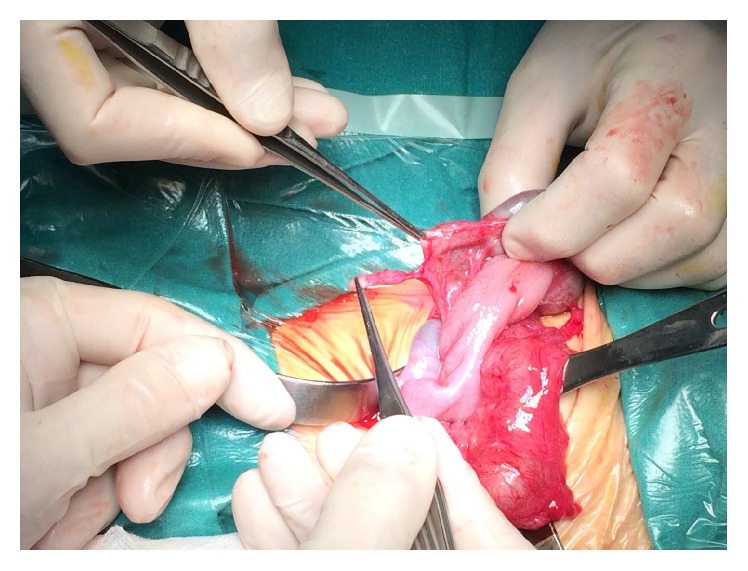


A staged approach was planned. On the first stage at referral, ascending and transverse colon appeared markedly dilated and four stenotic segments were identified: 1 in ascending and 3 in transverse colon (Figure 2), with no mesocolic defect. Moreover we documented an atresia of the middle appendix (Figure 3) and a normal small bowel length. Proximal resection and colostomy in the middle ascending colon were performed. Colonic biopsies excluded Hirschsprung's disease.

**Figure 4:**
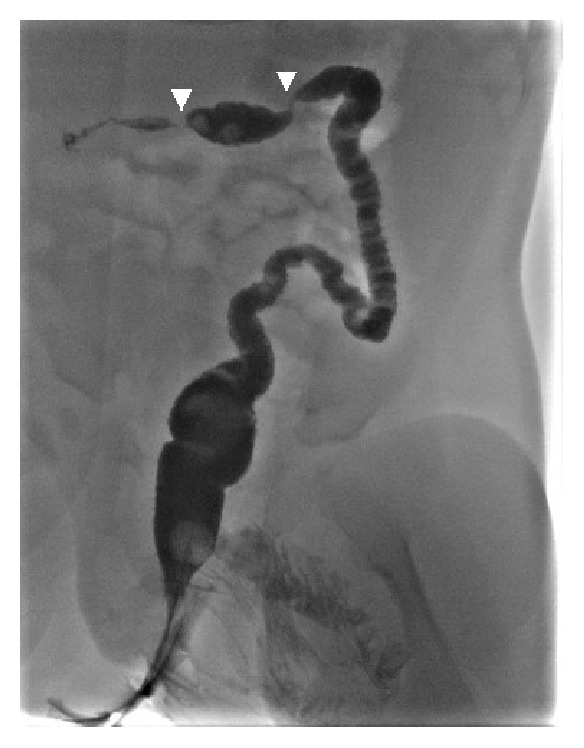


**Figure 5:**
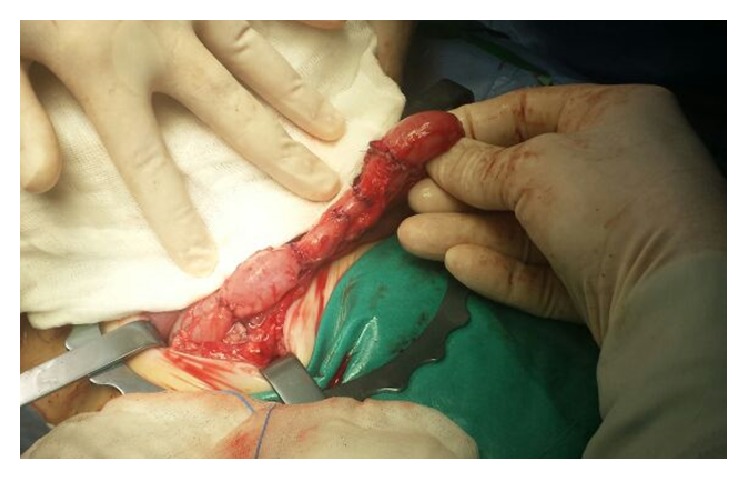


The child underwent a 3-month period of colonic nursing once a week to allow bowel preservation and lengthening before the final recanalization. A second contrast enema performed at 8 months of age showed the persistence of three stenotic segments at the transverse colon (Figure 4). A second laparotomy was thus performed. The three stenotic segments shown at contrast enema were identified; diameter discrepancy was significantly reduced (Figure 5). To avoid excessive colonic losses and iatrogenic malrotation following complete transverse colon resection, two enteroplasties at the first stenotic segment and a double resection and following colocolostomy were performed. The postoperative course was uneventful.

## 3. Discussion

Currently, colonic atresia is morphologically classified into four types, having both prognostic and therapeutic implications. The initial classification is based upon experimental study by Louw and Barnard in 1955, demonstrating intrauterine ischaemic insult as a possible mechanism leading to atresia and stenosis [15]. This hypothesis is nowadays accepted by many investigators [7, 9] and could also explain why intestinal atresia has been found to be associated with maternal smoking and vasoconstrictor drug exposure during pregnancy [16]. Moreover, defects in blood supply or areas of segmental ischemia due to embolus from the placenta, spontaneous thrombosis, or mechanical events such as volvulus or intussusceptions can explain the frequent association of atresia/stenosis with mesenteric defects.

Acquired stenosis is more common than congenital stenosis. Via the same mechanism of vascular compromise, the injured colon undergoes healing and scaring with narrowing of the affected intestine. Many authors investigated responsible pathogens. Pelizzo et al. [17] described three cases of isolated colon stenosis following* Norovirus* infection. In our case, neonatal serology could exclude the presence of any infection.

Congenital colonic stenosis could be considered as a further type of intestinal atresia. If we consider CCS alone, even in large case series [8, 11], it only counts for less than 1% of intestinal atresia. Excluding acquired stenosis following specific causative agents, 16 cases of CCS were identified from 1968 to nowadays. Age of presentation and localization of the stenosis are reported in Table 1.

Symptoms may be present at birth as constipation, abdominal pain, progressive abdominal distension, or failure to pass meconium within 48 hours but could also become manifest later in childhood as colicky abdominal pain, bilious vomiting, and abdominal distension. Baudet et al. [18] even reported a case of CCS diagnosed in adulthood presenting with abdominal pain, nausea, vomiting, and laxatives abuse.

According to the algorithm for the diagnostic imaging workup of the newborn with potential bowel obstruction by Maxfield et al. [19], the contrast enema performed orientates towards a colonic caliber change pattern with multiple narrowed segments. In these cases an elective, short-term laparotomy is required.

Differential diagnosis should include Hirschsprung's disease; colonic atresia is even reported to be associated with Hirschsprung's disease and missing the association before reconnecting the intestinal tract could lead to poor outcome and increased morbidity [20].

Surgical correction of colonic stenosis is the mainstay of therapy. Usually resection of atretic segment and primary anastomosis is the technique of choice in right-sided lesions [21], even if it necessitates sacrifice of whole proximal colon and ileocecal valve. In distal localization, many authors recommend a staged approach: initial colostomy and secondary resection and end-to-end anastomosis deferred for 9–12 months [22, 23]. This latter approach should be preferred; also when long segments of colon are involved, proximal segment is extremely dilated or distal mechanical or functional obstructions are present. Cox et al. [24] described as safe a primary anastomosis performed with a maximal diameter variance of 3 : 1 (proximal : distal). We thus planned a staged reconstruction with initial colostomy, weekly administration of warmed normal saline solution over a period of several months, and subsequent reconstruction. This procedure aimed to reduce diameter discrepancy [25], to avoid surgical and later functional problems, and to preserve colonic length.

The outcome of a patient with isolated colonic stenosis has improved significantly since Gross reported the death of a single patient in 1952 [26]. Improvement in resuscitation and perioperative care resulted in current survival rate of about 90%, if managed appropriately. The ultimate quality of life in this rare pediatric condition is influenced by eventual associated pathology and not by the presence of single or multiple colonic stenosis per se.

## 4. Conclusions

Considering both the literature and our case report, it is wise to evaluate CCS as a rare but possible cause of intestinal obstruction not only in the newborn but throughout the first years of life. In case of complex CCS, a planned staged approach is advisable. Early colostomy to allow a complete assessment and adaptation of distal segment and a delayed resection is recommended. The need for a careful exploration both proximally and distally is mandatory to exclude multiple stenosis or other associated gastrointestinal anomalies.

## Figures and Tables

**Table 1:** Congenital colonic stenosis: cases reported from 1968 to 2013.

Authors	Year	Patient	Age presentation	Localization of the stenosis
Saha et al. [2]	2013	1 male	1,5 y	Descending
Galván-Montaño et al. [3]	2010	1 male	3 y	Ascending
Ruggeri et al. [4]	2009	1 male	4 m	Ascending
Mizuno et al. [5]	2003	1 female	Newborn	Descending
García Vázquez et al. [6]	2002	1 male	2 m	Sigmoid
Abu-Judeh et al. [7]	2001	1 case	—	Ascending
Dalla Vecchia et al. [8]	1998	2 cases	Newborn	Not described
			Newborn	Not described
Sax [9]	1991	1 case	—	Descending-sigmoid
G. Pai and P. Pai et al. [10]	1990	1 female	4 m	Rectosigmoid junction
Rescorla and Grosfeld [11]	1985	1 case	—	Not described
			Newborn	Sigmoid
Schiller et al. [12]	1979	3 cases	Newborn	Descending
			Newborn	Sigmoid
Erskine [13]	1970	1 case	2 d	Descending-sigmoid
Benson et al. [14]	1968	1 case	—	Sigmoid
